# Dioscin induces demethylation of DAPK-1 and RASSF-1alpha genes via the antioxidant capacity, resulting in apoptosis of bladder cancer T24 cells

**DOI:** 10.17179/excli2016-571

**Published:** 2017-02-17

**Authors:** Qiang Zhou, Wei Song, Wei Xiao

**Affiliations:** 1 Department of Urology, Hunan Provincial People's Hospital, 61#, West Jiefang Road, Changsha, Hunan, P.R. China

**Keywords:** dioscin, methylation, DAPK-1, RASSF-1alpha, antioxidant capacity, bladder cancer

## Abstract

DNA methylation at CpG rich regions often occurs at tumor suppressor gene promoters, resulting in reduced gene expression and final carcinogenesis. Hypermethylation of tumor suppressor genes, including DAPK-1 and RASSF-1α genes, have been found in patients with bladder carcinoma (BC) in some western countries. Reactive oxygen species (ROS) was reported to play a causative role in gene hypermethylation. In this study, we detected the methylation status and expression of DAPK1 and RASSF-1α genes in tissue samples from Chinese BC patients, using methylation-specific PCR, reverse transcription PCR and western blotting. Further, we examined the ability of dioscin, a natural antioxidant, to regulate methylation status and expression of DAPK-1 and RASSF-1α genes in BC cell lines. In our results, DAPK-1 and RASSF-1α genes showed higher methylation level and lower express level in BC tissues than matched normal tissues. Treatment with dioscin decreased viability of BC 5637 and T24 cells, but not non-cancer bladder epithelial cell, SV-HUC-1. Dioscin triggered demethylation of DAPK1 and RASSF-1α genes in T24 cells and increased the gene and protein expression in 5637 and T24 cells. Both dioscin and substituted antioxidants (N-acetyl cysteine and Vitamin E) decreased intracellular ROS, but the effect of dioscin was abolished by adding H_2_O_2_. Similar to dioscin, the substituted antioxidants also induced the gene demethylation and T24 cell apoptosis. Co-treatment with dioscin and H_2_O_2_ had no such effects. Collectively, dioscin induces demethylation of DAPK-1 and RASSF-1α genes via the antioxidant capacity, resulting in apoptosis of bladder cancer T24 cells or inhibitory cell viability.

## Introduction

Bladder carcinoma (BC) is one of most common malignancy in the economically advanced countries, and the incident of BC disease has been increasing rapidly in some developing countries, like China, in recent years (Xu et al., 2016[16]; Christoph et al., 2006[1]). The pathogenesis of BC remains unclear, but recent bioinformatics analysis and epidemiological study indicate that abnormal hypermethylation in promoter regions of many tumor suppressor genes blocks their expression, which attenuates effects of their gene products against tumorigenesis, resulting in increased risk of BC occurrence (Christoph et al., 2006[2]; Jabłonowski et al., 2011[3]; Meng et al., 2012[5]). 

DAPK-1 (Death-associated protein kinase-1) and RASSF-1α (Ras-association domain family 1 isoform A) genes have been identified as important tumor suppressor gene, and down-regulation in their gene expression has been observed in various cancers (Christoph et al., 2006[1]). The product of the DAPK-1 gene is considered to be a positive mediator of apoptosis (Christoph et al., 2006[1]). One important mechanism by which tumor necrosis factor-α promotes apoptosis of tumor cells is through stimulating DAPK-1 functions (Turner-Brannen et al., 2011[13]). RASSF-1α gene exerts multiple actions against cancer formation and progression, via regulating cell death, cell cycle, and microtubule formation. An investigation undertaken in 42 patients of Poland with non-invasive BC found that hypermethylation in promoter regions of DAPK-1 gene accounts 64.3 % of patients (Jabłonowski et al., 2011[3]). In an independent study, using demethylating agents (5-Aza-2'-deoxycytidine and zebularine) restored DAPK-1 expression and effectively retarded growth of BC cells (Christoph et al., 2006[1]). Elevated promoter methylation of RASSF-1α gene was also observed in American BC patients by Meng et al. (2012[5]) who found that 73 % (30/41) of the transitional cell carcinoma, 100 % (3/3) of the squamous cell carcinoma, and 100 % (4/4) of the small cell carcinoma had the promoter methylation. The methylation ratio in BC tissues is much higher than that in normal adjacent tissues which only 6 % (1/16) of the methylation (Meng et al., 2012[5]). These data collectively suggest that promoter hypermethylation of DAPK1 and RASSF-1α genes is probably an important reason for their down-regulation in gene level, and is presumably associated with BC development and progression, whereas it is currently unknown about which reasons cause the promoter hypermethylation in BC. 

Reactive oxygen species (ROS) are a type of chemically reactive molecules containing oxygen, generated from both endogenous and exogenous origin. Abnormally elevated ROS has been closely linked with carcinogenesis, probably due to induction of epigenetic alterations (Wu and Ni, 2015[15]). Ongoing researches indicate that ROS contributes promoter hypermethylation of many genes, at least, though following three approaches:

1. ROS can function as catalysts of DNA methylation via induction of a series of chemical reactions;
2. Expression of DNA methyltransferases (DNMT) can be up-regulated by ROS, such as superoxide anion and hydrogen peroxide (H_2_O_2_);
3. DNA-induced DNA damage facilitates the recruitment of DNMT to DNA and the formation of DNMT-containing complexes (Wu and Ni, 2015[15]).

Given the promoting effect of ROS on gene promoter hypermethylation, using some antioxidants to scavenge ROS is presumably an effective method to attenuate or reverse the ROS-induced promoter hypermethylation and restore expression level of antioncogene, thereby repressing tumor growth and progression. 

Dioscin is a natural antioxidant widely present in some medicinal herbs. Pharmacological studies have demonstrated that dioscin has activity against various cancers, including cervical carcinoma, gastric cancer, lung cancer and colon cancer (Zhao et al., 2016[18]; Tong et al., 2014[12]; Wei et al., 2013[14]). But there is currently no study investigating impact of dioscin on BC. In addition, to our knowledge, no research has implicated the antioxidative activity of dioscin in regulation of gene methylation, and it is barely unknown about whether dioscin has the ability to store expression level of antioncogene that is methylated in promoter region, through anti-oxidative action. In this study, we detected the methylation status and expression of DAPK1 and RASSF-1α genes in tissue samples from Chinese BC patients, using methylation-specific PCR (MSP-PCR), reverse transcription PCR (RT-PCR) and western blotting. Further, we examined the ability of dioscin to regulate methylation status and expression of DAPK-1 and RASSF-1α genes in BC cell lines and the association of this ability with the anti-oxidative capacity. 

## Materials and Methods

### Tissue sample and ethics statement

BC and adjacent normal tissue samples were obtained from patients (Table 1(Tab. 1)) who underwent operation at the Third Xiangya Hospital of Central South University. The samples were frozen in liquid nitrogen immediately after surgery. All patients provided written informed consent in compliance with the code of ethics of the World Medical Association (Declaration of Helsinki; Ferney-Voltaire, France). This study was approved by the Ethics Committee of Xiangya School of Medicine (Changsha, People's Republic of China). 

### Cell culture and treatment

BC cell lines, including 5637 and T24 cells, and immortalized epithelial cell line of urinary bladder, SV-HUC-1 cells, were obtain from the American Type Culture Collection (ATCC, Manassas, VA, USA). All the cell lines were maintained at 37° C in RPMI-1640 medium (GibcoR; Life Technologies, Carlsbad, CA, USA) supplemented with 10 % fetal bovine serum (Invitrogen, Life Technologies, Carlsbad, CA, USA). These cells were treated with different doses of dioscin (0, 0.2, 1, 5 and 25 μg/mL) for 48 h. Besides, T24 cells were exposed to mixed antioxidants, containing 10 μg/mL N-acetyl cysteine and 20 μg/mL Vitamin E, or combination of 5 μg/mL dioscin and 5 μM/mL H_2_O_2_ for 48 h. All these agents were purchased from the Sigma Company (St. Louis, MO, USA), unless otherwise specified. 

### MSP-PCR analysis

MSP-PCR analysis was performed to determine methylation status of the promoter region of DAPK-1 and RASSF-1α genes in tissue samples and cell lines. Genomic DNA from tissue samples and cell lines was subjected to bisulfite modification using the CpGenome DNA Modification Kit (Invitrogen) according to the manufacturer's instructions. Primers were designed to locate on the 5'-upper region of each promoter, which can distinguish unmethylated (U) and methylated (M) alleles (Table 2(Tab. 2)). Cycling conditions were initial denaturation at 95˚C for 3 min, 40 cycles of 94° C for 30 sec, 65 (M) or 63° C (U) for 30 sec and 72° C for 30 sec. PCR products were separated on 4 % agarose gels, stained with ethidium bromide and visualized under UV illumination. 

### RT-PCR analysis

RT-PCR was performed to determine expression levels of DAPK-1 and RASSF-1α genes in indicated tissues and cells. RNA was isolated from the tissues and cells using TRIZOL (Takara Corp., Tokyo, Japan) according to the protocols supplied by the manufacturer, before RNA was reverse transcribed using the Reverse Transcription Kit (Applied Biosystems Inc., Foster City, CA, USA). The real-time PCR primers used to quantify DAPK-1, RASSF-1α and β-actin gene expression: 

DAPK-1 promoter forward,

5'-ATGATCCCACGTCAATCCAT-3' and DAPK-1 promoter reverse,

5'-CCACCAGGACAACTTGGAGT-3'; RASSF-1α promoter forward,

5'- GCAGGCATTGAGGAAGAGTC-3' and RASSF-1α promoter reverse,

5'- GTGCCCACATTCACACAGAC-3';

β-actin promoter forward,

5'- CATTAAGGAGAAGCTGTGCT-3' and β-actin promoter reverse,

5'- GTTGAAGGTAGTTTCGTGGA-3'. 

The real-time PCR was performed using FastStart SYBR-Green Master (cat. no. 04673484001) on a LightCycler480 system. The expression of DAPK-1 and RASSF-1α genes were normalized to that of endogenous β-actin gene.

### Western blot assay

DAPK-1 and RASSF-1α protein levels in indicated tissues and cells were evaluated by Western blot assay. Equal amounts (20 μg) of total protein extracted from the tissues and cells was loaded and separated on SDS-PAGE gels. Proteins were transferred to nitrocellulose membranes and subsequently immunoblotted with the primary antibodies, including anti-DAPK-1, anti-RASSF-1α, and anti-β-actin antibodies (Santa Cruz, CA, USA). Membranes were washed with TBS-T, and then incubated with secondary antibody. Proteins were detected using the Amersham enhanced chemiluminescence system (Pierce, Rockford, IL, USA) according to the manufacturer's instructions. The blots were visualized by enhanced chemiluminescence, using Fuji medical X-ray film (Fujiilm, Tokyo, Japan). 

### Cell viability assay

5637, T24 and SV-HUC-1 cells were seeded in 96-well plates in 100 μL complete medium and allowed to attach overnight. After the cells were subjected to diverse treatments, 3-(4,5-dimethyl-2-thiazolyl)-2,5-diphenyl-2H-tetrazolium bromide (MTT) (20 μL at 5 mg/mL) was added to each well. The medium was removed 4 h later and 150 μL dimethylsulfoxide was added subsequently to terminate the reaction. The plates were read with a microplate reader at 570 nm.

### Flow cytometry analysis of apoptotic cells

Apoptosis was detected using annexin V-FITC/propidium iodide (PI) staining followed by flow cytometry. After subjected to diverse treatments, T24 cells were incubated with annexin V-FITC and PI at room temperature for 15 min in the dark. The cells were collected and analyzed by a FACSCanto II flow cytometer (Becton Dickinson Immunocytometry System). 

### Intracellular ROS and reduced glutathione (GSH) measurement

The intracellular ROS level in cells was quantified using Reactive Oxygen Species Assay Kit (Beyotime, Nanjing, China). After washing with PBS, T24 cells were suspended in the 2′,7′-dichlorofluorescin diacetate (DCFH-DA) solution (10 μM) at 107/ml and incubated at 37° C for 20 min. Fluorescent intensity of DCFH-DA in the cells was detected by a fluorospectrophotometer (F-4000; Hitachi, Ltd., Tokyo, Japan). Glutathione Quantification Kit (Jianchen, Co., Nanjing, China) was used for the intracellular GSH measurement. This kit employs a fundamental reaction in which 5,59-dithiobis-2-nitrobenzoic acid (DTNB) and GSH react to generate 2-nitro-5-thiobenzoic acid and glutathione disulfide (GSSG). Because 2-nitro-5-thiobenzoic acid is a yellow product, the GSH concentration in the cell sample can be detected at 405 nm.

### Statistical analysis

Statistical analysis was performed using SPSS computer software (Version 12, SPSS, Chicago, USA). Statistical significance was determined using a two-tailed Student's t-test and standard deviations. All P-values < 0.05 were considered statistically significant.

## Results

### Methylation and expression of DAPK-1 and RASSF-1α genes in BC and adjacent normal tissues

**Figure 1:**
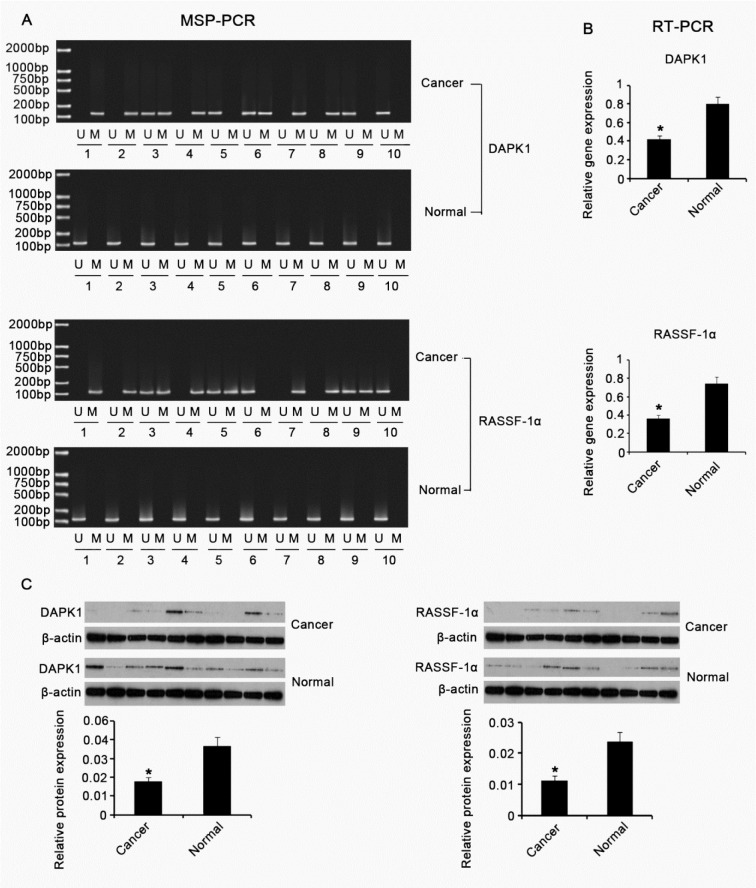
Methylation and expression of *DAPK-1* and RASSF-1α genes in BC and adjacent normal tissues BC tissues (n = 25) and adjacent normal tissues (n = 25) were collected and subjected to MSP-PCR (A), RT-PCR (B) and Western blot (C) assays to investigate methylation status and expression of* DAPK-1* and RASSF-1α genes in these tissues. U: unmethylated; M: methylated; *DAPK*1: Death-associated protein kinase-1; RASSF-1α: Ras-association domain family 1 isoform A. *P < 0.05 *vs.* control group.

BC tissues (n = 25) and adjacent normal tissues (n = 25) were collected and subjected to MSP-PCR to investigate promoter methylation status of DAPK-1 and RASSF-1α genes in these tissues. Each tissue sample experienced MSP-PCR test using both unmethylated (U) and methylated (M) primers. Figure 1A(Fig. 1) expresses the representative data. We found that the methylation frequencies of DAPK-1 and RASSF-1α genes in BC tissues are 64 % (15/25) and 80 % (20/25), respectively. In contrast, no methylation in promoter regions of DAPK-1 gene was detected in matched normal tissues, and only one case of the normal tissue sample showed the RASSF-1α gene methylation. Further, we examined the expression of DAPK-1 and RASSF-1α genes in the tissues using RT-PCR and Western blotting. As Figure 1B(Fig. 1) and 1C(Fig. 1) shown, DAPK-1 and RASSF-1α expressions in both gene and protein levels were significantly higher in BC tissues than in adjacent normal tissues (p < 0.05). 

### Effect of dioscin on viability of BC and marched normal cells 

**Figure 2:**
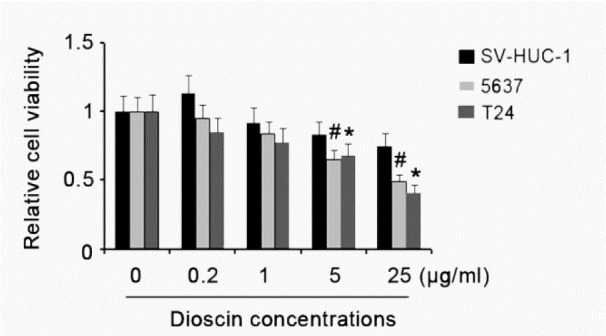
Effect of dioscin on viability of BC and marched normal cells BC cell lines, including 5637 and T24 cells, and immortalized epithelial cell line of urinary bladder, SV-HUC-1 cells, were treated with different doses of dioscin for 48h. Experiments were repeated at least three times. Each bar represents the mean of three independent experiments. ^#^*P* < 0.05 and *P < 0.05 vs. control group.

BC cell lines, including 5637 and T24 cells, and immortalized epithelial cell line of urinary bladder, SV-HUC-1 cells, were treated with different doses of dioscin for 48h. Cell viability test showed that dioscin caused a dose-dependent reduction in cell viability of 5637 and T24 cells (Figure 2(Fig. 2)). 5 and 25 μg/mL dioscin significantly decreased cell viability of both 5637 and T24 cells, as compared with corresponding untreated groups (p < 0.05). Except for 0.2 μg/mL dioscin, which slightly elevated the cell SV-HUC-1 viability, dioscin treatment induced different degrees of reductions in the cell viability. However, these reductions did not reach to statistical significance in despite of cell exposure to high concentrations of dioscin. 

### Effect of dioscin on methylation and expression of DAPK-1 and RASSF-1α genes in BC and marched normal cells

**Figure 3:**
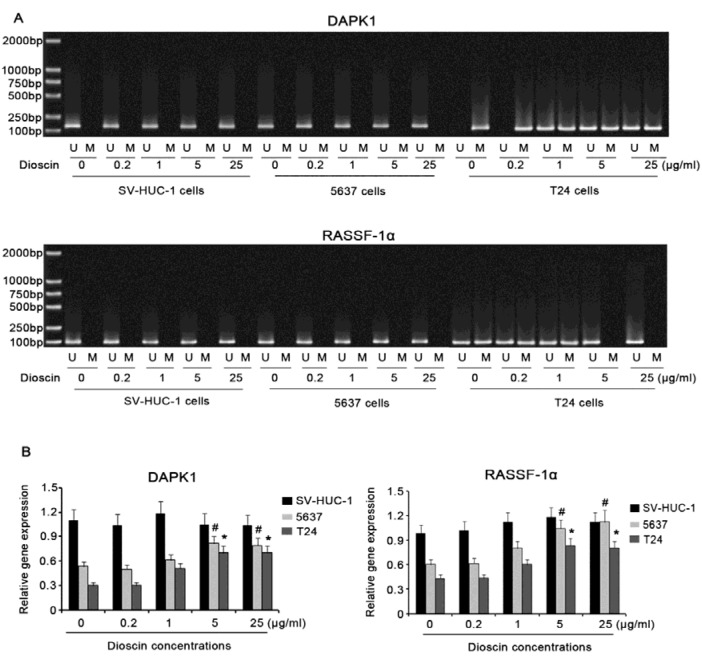
Effect of dioscin on methylation and expression of *DAPK-1* and RASSF-1α genes in BC and marched normal cells BC cell lines, including 5637 and T24 cells, and immortalized epithelial cell line of urinary bladder, SV-HUC-1 cells, were treated with different doses of dioscin for 48h. Then, MSP-PCR (A), RT-PCR (B) were performed to investigate methylation status and expression of* DAPK-1* and RASSF-1α genes in these cells. U: unmethylated; M: methylated; *DAPK*1: Death-associated protein kinase-1; RASSF-1α : Ras-association domain family 1 isoform A. Experiments were repeated at least three times. Each bar represents the mean of three independent experiments. ^#^*P* < 0.05 and *P < 0.05 vs. control group.

Results in Figure 3A(Fig. 3) indicated that there is not methylation in promoter regions of DAPK-1 and RASSF-1α genes in SV-HUC-1 and 5637 cells. Adding dioscin, regardless of concentrations, to the cells did not altered the unmethylated status. In contrast, DAPK-1 and RASSF-1α genes in T24 cells were methylated and hemi-methylated, respectively. Treatment of T24 cells with 5 or 25 μg/mL dioscin for 48h changed methylated status of DAPK-1 gene to hemi-methylated status. hemi-methylated RASSF-1α gene was switched to be unmethylated, after exposure to 5 or 25 μg/mL dioscin for 48h. RT-PCR was further performed to detect the alteration in DAPK-1 and RASSF-1α gene expression after the treatment with dioscin. When no dioscin was added to the cells, SV-HUC-1 cells showed higher DAPK-1 and RASSF-1α gene expression than 5637 and T24 cells. Supplement with dioscin had no significant effect on DAPK-1 and RASSF-1α gene expression in SV-HUC-1 cells, while 5 and 25 μg/mL dioscin raised DAPK-1 and RASSF-1α gene expression in both 5637 and T24 cells. 

### Antioxidant capacity of dioscin is associated with the regulation of DAPK-1 and RASSF-1α gene methylation in T24 cells 

**Figure 4:**
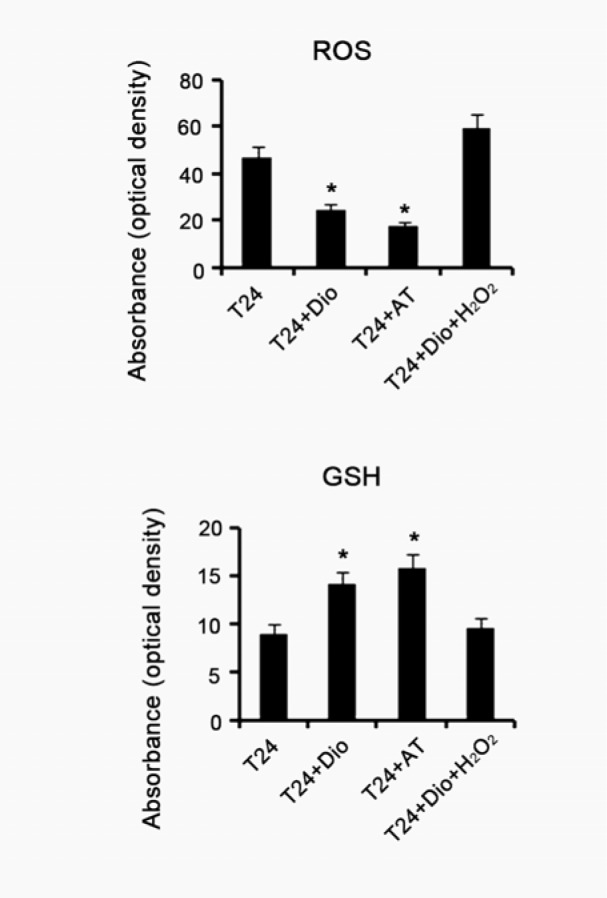
Intracellular ROS and GSH levels of BC T24 cells following diverse treatments. BC T24 cells were treated with 5μg/mL dioscin, mixed antioxidants (10 μg/mL N-acetyl cysteine and 20 μg/mL Vitamin E) or combination of 5μg/mL dioscin and 5 μM/mL H_2_O_2_ for 48h, followed by measurements of intracellular ROS and GSH levels. Dio: dioscin; AT: antioxidants composed of 10 μg/mL N-acetyl cysteine and 20 μg/mL Vitamin E; Dio+ H_2_O_2_: co-treatment with dioscin and H_2_O_2_. Experiments were repeated at least three times. Each bar represents the mean of three independent experiments. *P < 0.05 vs. control group.

**Figure 5:**
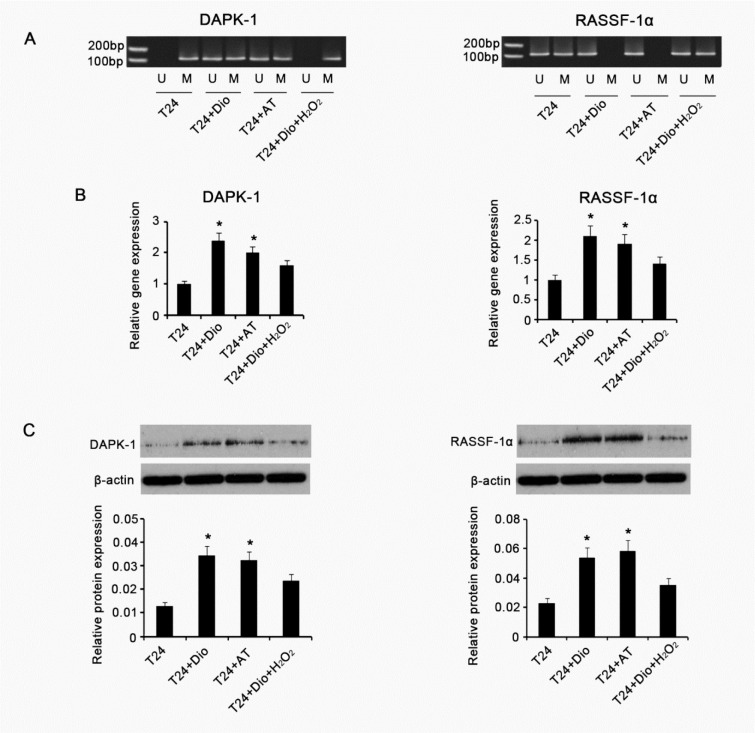
Methylation status and expression of* DAPK-1* and RASSF-1α genes in BC T24 cells following various treatments BC T24 cells were treated with 5μg/mL dioscin, mixed antioxidants (10 μg/mL N-acetyl cysteine and 20 μg/mL Vitamin E) or combination of 5μg/mL dioscin and 5 μM/mL H_2_O_2_ for 48h, followed by measurements of MSP-PCR (A), RT-PCR (B) and Western blot (C) assays to investigate methylation status and expression of* DAPK-1* and RASSF-1α genes in cells. U: unmethylated; M: methylated; *DAPK*1: Death-associated protein kinase-1; RASSF-1α : Ras-association domain family 1 isoform A. Dio: dioscin; AT: antioxidants composed of 10 μg/mL N-acetyl cysteine and 20 μg/mL Vitamin E; Dio+ H_2_O_2_: co-treatment with dioscin and H_2_O_2_. Experiments were repeated at least three times. Each bar represents the mean of three independent experiments. *P < 0.05 vs. control group.

To understand whether antioxidant capacity of dioscin is associated with the regulation of methylation status of DAPK-1 and RASSF-1α genes in T24 cells, we treated T24 cells with other antioxidants, which were consisted of 10 μg/mL N-acetyl cysteine and 20 μg/mL Vitamin E, or with combination of 5 μg/mL dioscin and 5 μM/mL H_2_O_2_. Examination of intracellular ROS showed that treatment with dioscin alone or the substituted antioxidants dramatically diminished ROS level, compared with control gourp (p < 0.05, Figure 4(Fig. 4)). Concurrent supplement with dioscin and H_2_O_2_ just caused a marginal elevation of intracellular ROS. GSH is an important endogenous antioxidant against ROS, thus it is commonly used as a mark to evaluate anti-/oxidative status in cells. It was found that intracellular GSH was notably increased after treatment with dioscin alone or the substituted antioxidants (p < 0.05). No significant alteration in GSH level was observed with concurrent supplement with dioscin and H_2_O_2_. MSP-PCR showed the substituted antioxidants changed methylated status of DAPK-1 gene to hemi-methylated status in T24 cells, as similar as the effect of dioscin (Figure 5A(Fig. 5)). H_2_O_2_ abrogated dioscin-induced alteration of DAPK-1 gene from methylated status to hemi-methylated status. hemi-methylated RASSF-1α gene is switched to be unmethylated by the substituted antioxidants. Combination of dioscin with H_2_O_2_ failed to lead to the alteration of hemi-methylated RASSF-1α gene to be unmethylated. We further assessed DAPK-1 and RASSF-1α expression in gene and protein levels via RT-PCR and Western blotting. As shown in Figure 5B(Fig. 5), both dioscin and substituted antioxidants increased DAPK-1 and RASSF-1α gene expression (p < 0.05). H_2_O_2_ abolished effect of dioscin on the up-regulation of DAPK-1 and RASSF-1α genes. DAPK-1 and RASSF-1α protein expression was increased with adding dioscin or substituted antioxidants to T24 cells (p < 0.05). Co-treatment with dioscin and H_2_O_2_, however, was unable to increased DAPK-1 and RASSF-1α protein expression. 

### Alteration of cell viability and apoptosis rate after various treatments 

**Figure 6:**
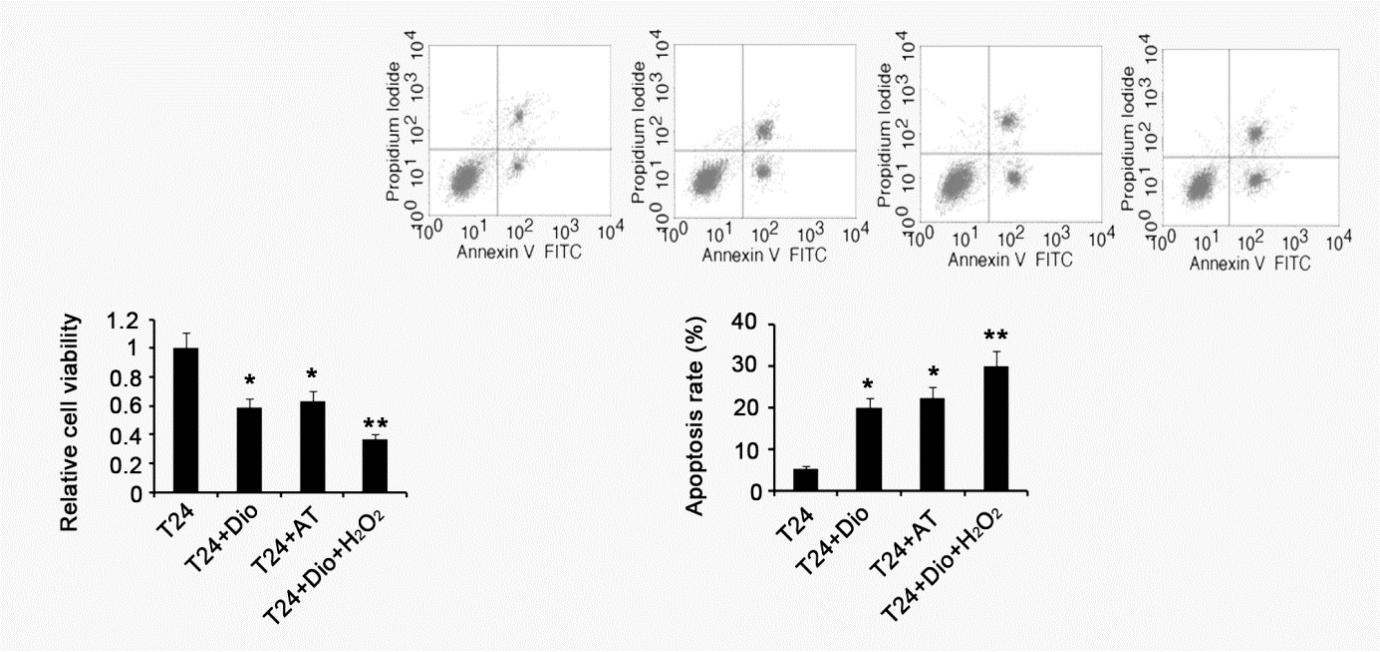
Alteration of cell viability and apoptosis rate after various treatments BC T24 cells were treated with 5μg/mL dioscin, mixed antioxidants (10 μg/mL N-acetyl cysteine and 20 μg/mL Vitamin E) or combination of 5μg/mL dioscin and 5 μM/mL H_2_O_2_ for 48h, followed by measurements of cell viability and apoptosis rate. Dio: dioscin; AT: antioxidants composed of 10 μg/mL N-acetyl cysteine and 20 μg/mL Vitamin E; Dio+ H_2_O_2_: co-treatment with dioscin and H_2_O_2_. Experiments were repeated at least three times. Each bar represents the mean of three independent experiments. *P < 0.05 and **P < 0.01 vs. control group.

Cell viability of T24 was significantly decreased by treatment with dioscin or substituted antioxidants, compared with control (p < 0.05), see Figure 6(Fig. 6). Co-treatment with dioscin and H_2_O_2_ further attenuated the cell viability (p < 0.01). Apoptosis rate was increased after treatment with dioscin or substituted antioxidants (p < 0.05). H_2_O_2_ enhanced the effect of dioscin on induction of apoptosis. 

## Discussion

BC is one the most frequent urogenital malignancies in developed counties and some developing countries with rapid economic development (Xu et al., 2016[16]; Christoph et al., 2006[1]). There is evidence that modern lifestyles have strong association with the high incident of BC, which can induce epigenetic transformation, resulting in aberrant expression of some cancer-related genes (Romagnolo et al., 2016[7]; Shankar et al., 2016[9]). Epigenetic control of gene transcription includes methylation of the DNA and covalent modifications, such as acetylation and methylation, of chromatin's histone proteins. Hypermethylation in promoter CpG islands and diminished expression of antioncogenes have been reported to be present in BC. Hypomethylation of the genomic DNA is relevant for gene silencing, leading to the functional inactivation. Previous researches have documented that hypermethylation of DAPK-1 and RASSF-1α genes was found in BC tissues from Poland and American patients, respectively (Jabłonowski et al., 2011[3]; Meng et al., 2012[5]). Similarly, this study revealed that promoter methylation levels of DAPK-1 and RASSF-1α genes in BC tissues from Chinese patients were significantly elevated when compared to those in adjacent normal tissues. These studies imply that hypermethylation of DAPK-1 and RASSF-1α genes is probably a common event in BC patients worldwide. Further, we uncovered that DAPK-1 and RASSF-1α expression in both gene and protein levels were notably decreased in BC tissues, compared with normal bladder tissues. Thus, we suggest that hypermethylation of DAPK-1 and RASSF-1α genes is likely associated with their down-regulation. 

Dioscin is a natural antioxidant, exerting anti-cancer activity, but the effect of dioscin has never been investigated in BC. In this study, we found that dioscin notably decreased viability of BC 5637 and T24 cells, but marginally inhibited viability of bladder non-cancer cells (SV-HUC-1). These data indicated that dioscin has preferential growth inhibitory activity on BC cells than non-cancer bladder cells. One possible reason is that dioscin could up-regulate DAPK-1 and RASSF-1α expression in 5637 and T24 cells, but not in SV-HUC-1 cells, as observed in our finding. DAPK-1 and RASSF-1α genes play important role in growth inhibition and apoptosis of tumor cells. DAPK1 is a kinase involved in the p53-dependent apoptosis pathway and the modulation of apoptosis initiated by several death-inducers such as TNF-α and INF-γ (Tian et al., 2015[11]; Kwon et al., 2016[4]). In various cancers, including ovarian endometriosis and endometrioid carcinoma, non-small cell lung cancer and cervical cancer, DAPK1 is down-regulated and thereby contributes to reduced sensitivity to apoptotic signal and increased tumor cell survival and proliferation, causing cancer development and recurrence (Christoph et al., 2006[1]; Tian et al., 2015[11]; Kwon et al., 2016[4]). RASSF1α promoter methylation has been used as a biomarker for detecting field cancerization in non-invasive breast cancer and as a prognostic factor in invasive breast cancer (Spitzwieser et al., 2015[10]; Shakeri et al., 2016[8]). Previous studies have documented lower expression of RASSF1α in non-small cell lung cancer, esophageal squamous cell carcinoma and lacrimal gland carcinoma (Zeng et al., 2015[17]). Patients with lower RASSF1α expression showed a higher recurrence probability and unfavorable prognosis (Zeng et al., 2015[17]). Restoration of RASSF1α expression significantly inhibited gastric cancer cell viability, colony formation and induced the cell cycle arrest and apoptosis (Zhou et al., 2015[19]).

Over-production of ROS has been closely linked with gene hypomethylation, although the underlying mechanisms are incompletely understood. Zhou et al (2016[20]) found that long-term exposure to PM2.5, namely the fine particulate matter, suppressed p53 expression in lung cancer cells, via ROS-Akt-DNMT3B pathway-mediated promoter hypermethylation. In addition, exposure to low-dose Fe-ion radiation causes ROS production, further resulting in increased DNA methylation in mouse bone marrow hematopoietic progenitor and stem cells (Miousse et al., 2014[6]). Although it is unclear about whether the hypermethylation of DAPK-1 and RASSF-1α genes in BC cells is correlated with increased ROS, our study showed that treatment of BC T24 cells with dioscin induced the gene demethylation. Similar outcome was also observed with the substitute antioxidants composed of N-acetyl cysteine and Vitamin E. More importantly, concurrent treatment with a oxidant, H_2_O_2_, abolished the dioscin anti-oxidative capacity and the effect on the gene demethylation. These evidence indicated that ability of dioscin to gene demethylation is associated with the anti-oxidative capacity. 

In T24 cells, promoter demethylation of DAPK-1 and RASSF-1α genes induced by both dioscin and the antioxidants is accompanied with the gene up-regulation. H_2_O_2_ abolished the effect of dioscin on the gene demethylation, which reversed DAPK-1 and RASSF-1α expression that is enhanced by dioscin. These data suggest that dioscin up-regulating DAPK-1 and RASSF-1α expression is dependent of the demethylation action. Unlikely, there is no methylation in promoter regions of DAPK-1 and RASSF-1α genes in BC 5637 cells, thus the up-regulated DAPK-1 and RASSF-1α expression induced by dioscin in the cells is probably not related to the demethylation activity. Further study is needed to clarify the mechanism by which dioscin up-regulated DAPK-1 and RASSF-1α expression in BC 5637 cells. An unexpected result is that co-treatment with dioscin and H_2_O_2_ had a better effect on reduction of cell viability and induction of cell apoptosis than treatment with dioscin alone, even though H_2_O_2_ abrogated effect of dioscin on the demethylation of DAPK-1 and RASSF-1α genes. It should be noted that H_2_O_2_ originally has strong ability to induce cell apoptosis through causing cell damages in cell membrane and DNA and stimulating multiple apoptotic signal pathways, which may explain the result. 

In summary, this study provided direct evidence that dioscin suppresses viability of BC 5637 and T24 cells and increases the apoptosis rate, via the up-regulation of DAPK1 and RASSF-1α genes. More importantly, we verified that dioscin-induced up-regulation of DAPK1 and RASSF-1α genes in BC T24 cells is related to the induction of gene demethylation by the anti-oxidative activity. 

## Conflict of interest

The authors declare no conflict of interest.

## Figures and Tables

**Table 1:**
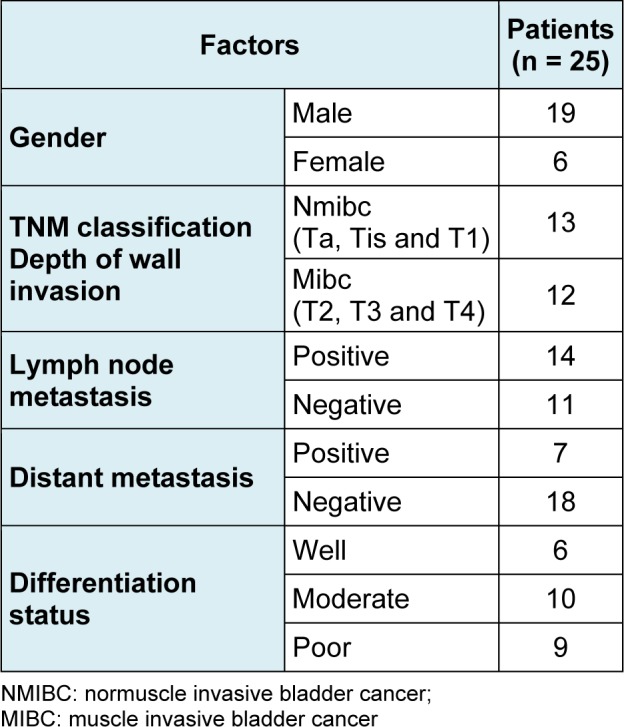
Clinicopathological characteristics of BC patients

**Table 2:**
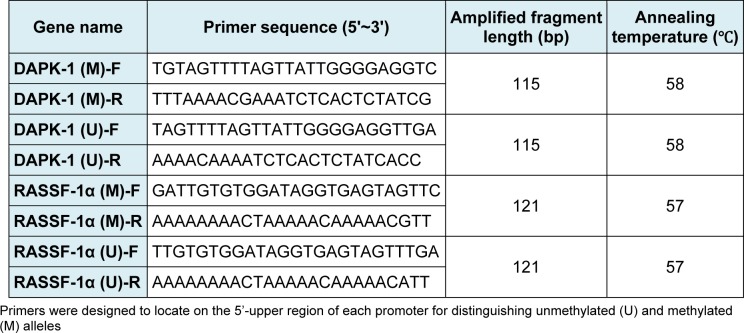
Primers sequence for MSP-PCR analysis
